# *catena*-Poly[[[iodidocopper(I)]-{μ-*N*-[(pyridin-2-yl-κ*N*)methyl­idene]pyridin-3-amine-κ^2^ *N* ^3^:*N* ^1^}] acetonitrile hemisolvate]

**DOI:** 10.1107/S1600536812037270

**Published:** 2012-09-19

**Authors:** Ali Mahmoudi, Saeed Dehghanpour, Mojtaba Babakhodaverdi

**Affiliations:** a Department of Chemistry, Islamic Azad University, Karaj Branch, Karaj, Iran; b Department of Chemistry, Alzahra University, Tehran, Iran

## Abstract

In the asymmetric unit of the title polymeric complex, {[CuI(C_11_H_9_N_3_)]·0.5CH_3_CN}_*n*_, there are two Cu^I^ atoms, two *N*-[(pyridin-2-yl-κ*N*)methyl­idene]pyridin-3-amine (PyPy) ligands and two I atoms. Both Cu^I^ atoms have a distorted tetra­hedral geometry, each being coordinated by one I atom, two N atoms of one PyPy ligand and one N atom from an adjacent PyPy ligand. In the crystal, infinite helical chains of [Cu_2_(PyPy)_2_]_*n*_ are formed propagating along the *b* axis. These chains are linked *via* weak C—H⋯I hydrogen bonds and π–π stacking inter­actions [shortest centroid–centroid distance = 3.2727 (14) Å]. During the refinement, electron-density peaks were located that were believed to be highly disordered solvent mol­ecules (possibly acetonitrile). The SQUEEZE option in *PLATON* [Spek (2009 ▶). *Acta Cryst*. D**65**, 148–155] indicated there were solvent cavities with a total volume of 196 Å^3^ containing approximately 60 electrons per unit cell, which equated to one mol­ecule of acetonitrile per asymmetric unit.

## Related literature

For related structures and applications of coordination polymers, see: Moulton & Zaworotko (2001 ▶); Fei *et al.* (2000 ▶). For the synthesis of the title ligand, see: Dehghanpour *et al.* (2009 ▶). 
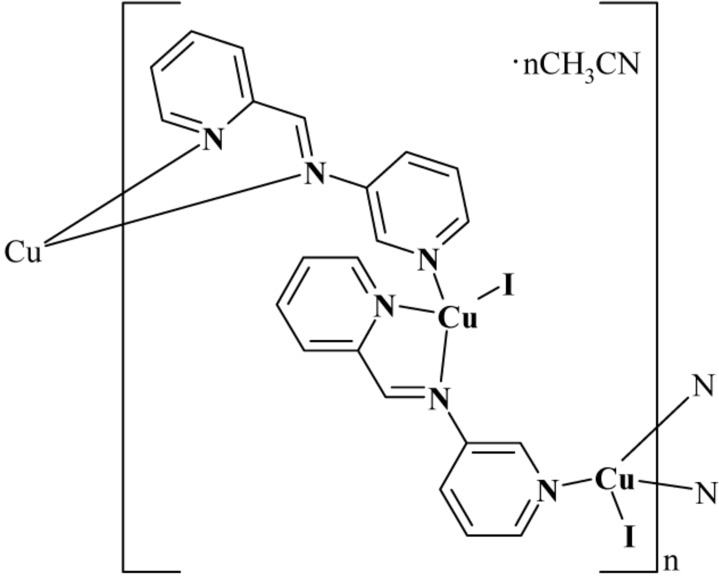


## Experimental

### 

#### Crystal data

[CuI(C_11_H_9_N_3_)]·0.5C_2_H_3_N
*M* *_r_* = 394.18
Monoclinic, 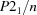

*a* = 7.1800 (2) Å
*b* = 13.2303 (7) Å
*c* = 27.9383 (13) Å
β = 90.741 (3)°
*V* = 2653.7 (2) Å^3^
*Z* = 8
Mo *K*α radiation
μ = 3.96 mm^−1^
*T* = 150 K
0.17 × 0.12 × 0.10 mm

#### Data collection

Nonius KappaCCD diffractometer
Absorption correction: multi-scan (*SORTAV*; Blessing, 1995 ▶) *T*_min_ = 0.569, *T*_max_ = 0.733
19063 measured reflections
4676 independent reflections
2627 reflections with *I* > 2σ(*I*)
*R*_int_ = 0.104

#### Refinement

*R*[*F* ^2^ > 2σ(*F* ^2^)] = 0.060
*wR*(*F* ^2^) = 0.178
*S* = 1.02
4676 reflections
289 parameters
H-atom parameters constrained
Δρ_max_ = 1.39 e Å^−3^
Δρ_min_ = −1.24 e Å^−3^

### 

Data collection: *COLLECT* (Nonius, 2002 ▶); cell refinement: *DENZO-SMN* (Otwinowski & Minor, 1997 ▶); data reduction: *DENZO-SMN*; program(s) used to solve structure: *SIR92* (Altomare *et al.*, 1994 ▶); program(s) used to refine structure: *SHELXL97* (Sheldrick, 2008 ▶); molecular graphics: *PLATON* (Spek, 2009 ▶); software used to prepare material for publication: *SHELXTL* (Sheldrick, 2008 ▶).

## Supplementary Material

Crystal structure: contains datablock(s) I, global. DOI: 10.1107/S1600536812037270/su2480sup1.cif

Structure factors: contains datablock(s) I. DOI: 10.1107/S1600536812037270/su2480Isup2.hkl

Additional supplementary materials:  crystallographic information; 3D view; checkCIF report

## Figures and Tables

**Table 1:** Hydrogen-bond geometry (Å, °)

*D*—H⋯*A*	*D*—H	H⋯*A*	*D*⋯*A*	*D*—H⋯*A*
C22—H22*A*⋯I1^i^	0.95	3.03	3.789 (12)	138
C20—H20*A*⋯I1^ii^	0.95	3.14	4.025 (12)	156
C17—H17*A*⋯I2^iii^	0.95	3.16	4.011 (11)	149

Symmetry codes: (i) 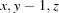; (ii) 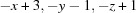; (iii) 
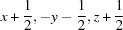
.

**Fig. 1.**
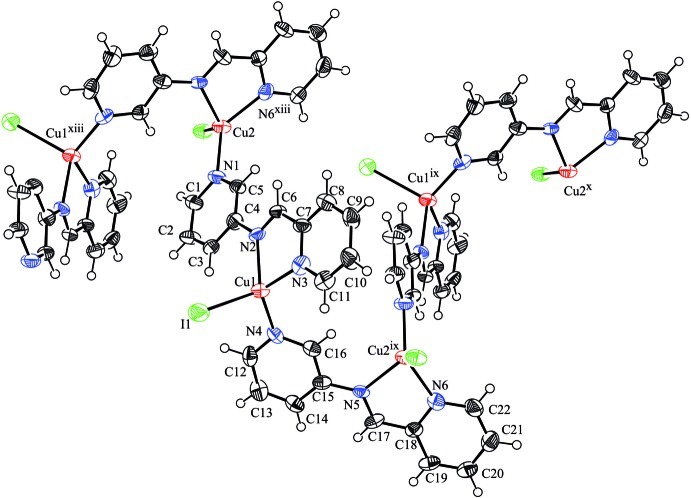
A view of the molecular structure of the title complex, with atom numbering. Displacement ellipsoids are drawn at the 50% probability level [Symmetry codes: (ix) 5/2 - x, -1/2 + y, 1/2 - z; (x) x, -1 + y, z; (xiii) 5/2 - x, 1/2 + y, 1/2 - z].

**Fig. 2.**
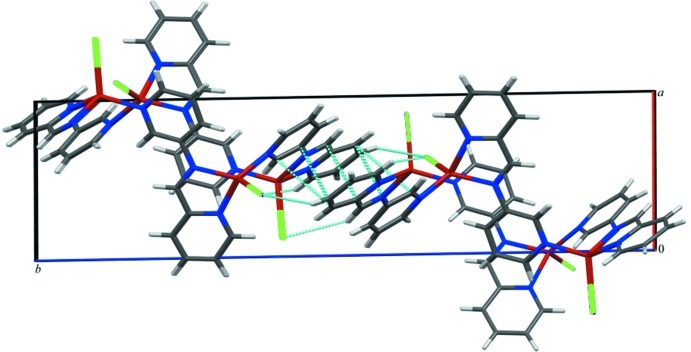
A view of the π–π interactions and C—H···I hydrogen bonds (dotted lines) in the crystal structure of the title compound.

## supplementary crystallographic information

### Comment

In recent years, coordination polymers have received much attention due to their variety of architectures and the potential applications as functional materials (Moulton & Zaworotko, 2001). Early reports have shown that nitrogen heterocyclic ligands have been employed in the synthesis of many novel structures (Fei *et al.*, 2000). Here, we report on the synthetic and crystal structure of a novel copper iodide complex based on the ligand pyridin-3-ylpyridin-2-ylmethyleneamine (PyPy).

The asymmetric unit of the title compound, Fig. 1, contains two Cu^I^ atoms, two pyridin-3-ylpyridin-2-ylmethyleneamine (Dehghanpour *et al.*, 2009) ligands, and two I atoms, Each Cu^+^ atom is four-coordinated in a distorted tetrahedral configuration by two N atoms from one PyPy ligand, one N atom from an adjacent PyPy ligand and one I atom. Each PyPy ligand chelates the Cu atom (*via* N, N' atoms) and also bridges to another Cu atom (with N" atom), resulting in the formation of chains propagating along the *b* axis.

The two ligands in the asymmetric unit are nearly planar. In ligand A the interplanar angles between chelate ring (N2/C6/C7/N3) and pyridine ring (lN3/C7-C11) is 2.11 (3)°, while for ligand B [chelate ring N5/C17/C18/N6 and pyridine ring N6/C18-C22] the same angle is 5.82 (4)°. In ligand A the two pyridine rings (N1/C1-C5 and N3/C7-C11) are inclined to one another by 12.11 (4)°. In ligand B the two pyridine rings (N6/C18-C22 and N4/C12-5C16) are inclined to one another by 7.49 (3)°. However, the interplanar angle between two ligand mean planes (A and B) is 52.82 (1)°.

In the crystal, these chains interact *via*π–π interactions between adjacent, inversion replated PyPy ligands The shortest distance of 3.2727 (14) Å [C15-C19 ring, symmetry code: (iii) = -*x* + 3, -*y* - 1, -*z* + 1] is observed between two inversion related ligands. These chains are further connected through C—H···I interactions (Table 1 and Fig 2.).

### Experimental

The title complex was prepared by the reaction of CuI (19.1 mg, 0.1 mmol) and pyridin-3-ylpyridin-2-ylmethyleneamine (18.3 mg, 0.1 mmol) in 20 ml of acetonitrile at room temperature. Crystals of the title compound, suitable for X-ray analysis, were obtained by slow evaporation of the solvent at rt.

### Refinement

H atoms were placed in calculated positions and included in the refinement in a riding-motion approximation: C—H = 0.95 Å with *U*_iso_(H)= 1.2*U*_eq_(C). During the refinement of the structure, electron density peaks were located that were believed to be highly disordered solvent molecules (possibly acetonitrile). Attempts to model the solvent molecule were not successful. The SQUEEZE option in PLATON (Spek, A. L. (2009). Acta Cryst. D65, 148-155) indicated there were solvent cavities with a total volume of 196 Å^3^ containing approximately 60 electrons per unit cell. This was equated to one molecule of acetonitrile per asymmetric unit. The density, the F(000) value, the molecular weight and the formula are given taking into account the results obtained with the SQUEEZE option in PLATON.

### Crystal data

**Table:**

[CuI(C_11_H_9_N_3_)]·0.5C_2_H_3_N	*F*(000) = 1512
*M**_r_* = 394.18	*D*_x_ = 1.973 Mg m^−^^3^
Monoclinic, *P*2_1_/*n*	Mo *K*α radiation, λ = 0.71073 Å
Hall symbol: -P 2yn	Cell parameters from 19063 reflections
*a* = 7.1800 (2) Å	θ = 2.5–25.0°
*b* = 13.2303 (7) Å	µ = 3.96 mm^−^^1^
*c* = 27.9383 (13) Å	*T* = 150 K
β = 90.741 (3)°	Block, brown
*V* = 2653.7 (2) Å^3^	0.17 × 0.12 × 0.10 mm
*Z* = 8	

### Data collection

**Table:**

Nonius KappaCCD diffractometer	4676 independent reflections
Radiation source: fine-focus sealed tube	2627 reflections with *I* > 2σ(*I*)
Graphite monochromator	*R*_int_ = 0.104
Detector resolution: 9 pixels mm^-1^	θ_max_ = 25.0°, θ_min_ = 2.7°
φ scans and ω scans with κ offsets	*h* = −8→8
Absorption correction: multi-scan (*SORTAV*; Blessing, 1995)	*k* = −15→15
*T*_min_ = 0.569, *T*_max_ = 0.733	*l* = −33→33
19063 measured reflections	

### Refinement

**Table:**

Refinement on *F*^2^	Primary atom site location: structure-invariant direct methods
Least-squares matrix: full	Secondary atom site location: difference Fourier map
*R*[*F*^2^ > 2σ(*F*^2^)] = 0.060	Hydrogen site location: inferred from neighbouring sites
*wR*(*F*^2^) = 0.178	H-atom parameters constrained
*S* = 1.02	*w* = 1/[σ^2^(*F*_o_^2^) + (0.0943*P*)^2^] where *P* = (*F*_o_^2^ + 2*F*_c_^2^)/3
4676 reflections	(Δ/σ)_max_ = 0.002
289 parameters	Δρ_max_ = 1.39 e Å^−^^3^
0 restraints	Δρ_min_ = −1.24 e Å^−^^3^

### Special details

**Table:**

Geometry. All e.s.d.'s (except the e.s.d. in the dihedral angle between two l.s. planes) are estimated using the full covariance matrix. The cell e.s.d.'s are taken into account individually in the estimation of e.s.d.'s in distances, angles and torsion angles; correlations between e.s.d.'s in cell parameters are only used when they are defined by crystal symmetry. An approximate (isotropic) treatment of cell e.s.d.'s is used for estimating e.s.d.'s involving l.s. planes.
Refinement. Refinement of *F*^2^ against ALL reflections. The weighted *R*-factor *wR* and goodness of fit *S* are based on *F*^2^, conventional *R*-factors *R* are based on *F*, with *F* set to zero for negative *F*^2^. The threshold expression of *F*^2^ > σ(*F*^2^) is used only for calculating *R*-factors(gt) *etc*. and is not relevant to the choice of reflections for refinement. *R*-factors based on *F*^2^ are statistically about twice as large as those based on *F*, and *R*- factors based on ALL data will be even larger.

### Fractional atomic coordinates and isotropic or equivalent isotropic displacement parameters (Å^2^)

**Table:**

	*x*	*y*	*z*	*U*_iso_*/*U*_eq_	
I1	1.60988 (13)	−0.00459 (7)	0.36787 (3)	0.0589 (3)	
I2	0.61501 (9)	−0.05499 (6)	0.10018 (2)	0.0389 (3)	
Cu1	1.50363 (18)	−0.17127 (11)	0.32943 (4)	0.0400 (4)	
Cu2	0.97229 (16)	−0.07450 (11)	0.10723 (4)	0.0365 (4)	
N1	1.0526 (12)	−0.0684 (7)	0.1767 (3)	0.039 (2)	
N2	1.4312 (11)	−0.1559 (6)	0.2576 (3)	0.032 (2)	
N3	1.7228 (11)	−0.2492 (7)	0.2989 (3)	0.039 (2)	
N4	1.3514 (11)	−0.2392 (7)	0.3785 (3)	0.036 (2)	
N5	1.3643 (10)	−0.4852 (7)	0.4384 (3)	0.029 (2)	
N6	1.4522 (11)	−0.6811 (7)	0.4423 (3)	0.037 (2)	
C1	0.9411 (16)	−0.0224 (9)	0.2091 (4)	0.042 (3)	
H1A	0.8262	0.0063	0.1987	0.051*	
C2	0.9913 (15)	−0.0165 (9)	0.2567 (4)	0.044 (3)	
H2A	0.9147	0.0176	0.2790	0.053*	
C3	1.1567 (14)	−0.0616 (8)	0.2713 (4)	0.038 (3)	
H3A	1.1948	−0.0593	0.3040	0.045*	
C4	1.2640 (14)	−0.1092 (8)	0.2385 (4)	0.037 (3)	
C5	1.2109 (13)	−0.1129 (8)	0.1925 (3)	0.030 (2)	
H5A	1.2867	−0.1478	0.1703	0.036*	
C6	1.5589 (15)	−0.1921 (8)	0.2304 (4)	0.036 (3)	
H6A	1.5464	−0.1853	0.1966	0.043*	
C7	1.7202 (14)	−0.2428 (8)	0.2502 (3)	0.034 (3)	
C8	1.8553 (15)	−0.2858 (9)	0.2230 (4)	0.042 (3)	
H8A	1.8478	−0.2806	0.1891	0.050*	
C9	2.0017 (16)	−0.3364 (9)	0.2440 (4)	0.044 (3)	
H9A	2.0955	−0.3666	0.2250	0.053*	
C10	2.0096 (15)	−0.3424 (9)	0.2930 (4)	0.049 (3)	
H10A	2.1101	−0.3757	0.3088	0.059*	
C11	1.8675 (14)	−0.2987 (9)	0.3186 (4)	0.045 (3)	
H11A	1.8725	−0.3041	0.3525	0.054*	
C12	1.2362 (14)	−0.1885 (9)	0.4062 (4)	0.040 (3)	
H12A	1.2122	−0.1196	0.3988	0.049*	
C13	1.1467 (14)	−0.2310 (9)	0.4462 (4)	0.045 (3)	
H13A	1.0588	−0.1928	0.4638	0.054*	
C14	1.1884 (13)	−0.3277 (9)	0.4593 (4)	0.040 (3)	
H14A	1.1356	−0.3570	0.4870	0.048*	
C15	1.3101 (13)	−0.3826 (9)	0.4310 (3)	0.033 (3)	
C16	1.3879 (12)	−0.3373 (8)	0.3914 (3)	0.031 (3)	
H16A	1.4704	−0.3758	0.3724	0.037*	
C17	1.2990 (12)	−0.5374 (8)	0.4725 (4)	0.033 (3)	
H17A	1.2150	−0.5068	0.4941	0.040*	
C18	1.3492 (14)	−0.6418 (8)	0.4791 (4)	0.035 (3)	
C19	1.3045 (14)	−0.6992 (9)	0.5186 (4)	0.042 (3)	
H19A	1.2341	−0.6695	0.5435	0.051*	
C20	1.3595 (14)	−0.7977 (10)	0.5228 (4)	0.043 (3)	
H20A	1.3260	−0.8378	0.5496	0.052*	
C21	1.4685 (16)	−0.8371 (9)	0.4854 (4)	0.050 (3)	
H21A	1.5134	−0.9045	0.4872	0.060*	
C22	1.5098 (16)	−0.7777 (9)	0.4462 (4)	0.049 (3)	
H22A	1.5814	−0.8060	0.4212	0.058*	

### Atomic displacement parameters (Å^2^)

**Table:**

	*U*^11^	*U*^22^	*U*^33^	*U*^12^	*U*^13^	*U*^23^
I1	0.0911 (7)	0.0473 (6)	0.0383 (5)	−0.0199 (5)	−0.0001 (4)	−0.0023 (4)
I2	0.0311 (4)	0.0564 (6)	0.0292 (4)	0.0023 (3)	−0.0015 (3)	0.0035 (4)
Cu1	0.0506 (8)	0.0436 (9)	0.0259 (7)	0.0010 (6)	0.0002 (6)	0.0026 (6)
Cu2	0.0346 (7)	0.0508 (10)	0.0241 (7)	0.0002 (6)	0.0001 (5)	0.0010 (6)
N1	0.048 (6)	0.047 (6)	0.023 (5)	0.004 (5)	−0.001 (4)	−0.002 (4)
N2	0.037 (5)	0.034 (6)	0.024 (5)	0.001 (4)	−0.005 (4)	0.007 (4)
N3	0.031 (5)	0.039 (6)	0.046 (6)	−0.005 (4)	−0.009 (4)	0.003 (5)
N4	0.033 (5)	0.040 (6)	0.034 (5)	0.006 (4)	0.000 (4)	−0.012 (5)
N5	0.029 (4)	0.041 (6)	0.019 (4)	0.004 (4)	−0.005 (3)	−0.003 (4)
N6	0.035 (5)	0.036 (6)	0.038 (5)	−0.010 (4)	−0.007 (4)	−0.008 (5)
C1	0.051 (7)	0.046 (8)	0.030 (6)	0.006 (6)	0.004 (5)	−0.003 (5)
C2	0.044 (7)	0.065 (9)	0.024 (6)	0.006 (6)	0.004 (5)	0.001 (6)
C3	0.047 (7)	0.037 (7)	0.029 (6)	0.013 (5)	−0.009 (5)	0.005 (5)
C4	0.036 (6)	0.037 (7)	0.037 (7)	0.005 (5)	−0.003 (5)	0.000 (6)
C5	0.030 (6)	0.036 (7)	0.025 (6)	0.000 (5)	−0.004 (4)	−0.003 (5)
C6	0.055 (7)	0.034 (7)	0.019 (5)	−0.003 (5)	−0.001 (5)	0.002 (5)
C7	0.042 (6)	0.038 (7)	0.022 (6)	−0.005 (5)	−0.004 (5)	−0.001 (5)
C8	0.044 (7)	0.047 (8)	0.034 (6)	−0.009 (6)	0.005 (5)	−0.007 (6)
C9	0.049 (7)	0.037 (7)	0.046 (8)	0.003 (6)	−0.017 (6)	−0.014 (6)
C10	0.040 (7)	0.052 (9)	0.054 (8)	0.018 (6)	−0.004 (6)	−0.007 (7)
C11	0.040 (7)	0.048 (8)	0.045 (7)	−0.006 (6)	−0.004 (5)	0.005 (6)
C12	0.037 (6)	0.031 (7)	0.053 (8)	0.006 (5)	−0.008 (5)	−0.005 (6)
C13	0.031 (6)	0.043 (8)	0.061 (8)	−0.003 (5)	0.008 (5)	−0.007 (6)
C14	0.036 (6)	0.047 (8)	0.037 (6)	−0.005 (5)	0.006 (5)	−0.009 (6)
C15	0.028 (5)	0.047 (8)	0.025 (6)	−0.001 (5)	−0.008 (4)	0.002 (5)
C16	0.025 (5)	0.039 (7)	0.030 (6)	−0.011 (5)	−0.005 (4)	−0.008 (5)
C17	0.020 (5)	0.048 (8)	0.031 (6)	−0.005 (5)	−0.006 (4)	0.013 (5)
C18	0.038 (6)	0.038 (7)	0.028 (6)	−0.008 (5)	−0.014 (5)	0.000 (5)
C19	0.042 (6)	0.055 (9)	0.030 (6)	−0.010 (6)	0.004 (5)	0.011 (6)
C20	0.043 (7)	0.052 (9)	0.035 (7)	−0.006 (6)	−0.008 (5)	0.008 (6)
C21	0.062 (8)	0.033 (8)	0.054 (8)	−0.008 (6)	−0.019 (6)	−0.002 (6)
C22	0.068 (8)	0.038 (8)	0.040 (7)	−0.010 (6)	0.004 (6)	−0.008 (6)

### Geometric parameters (Å, º)

**Table:**

I1—Cu1	2.5645 (16)	C5—H5A	0.9500
I2—Cu2	2.5832 (14)	C6—C7	1.443 (14)
Cu1—N4	1.980 (9)	C6—H6A	0.9500
Cu1—N3	2.074 (9)	C7—C8	1.365 (14)
Cu1—N2	2.078 (8)	C8—C9	1.372 (15)
Cu2—N1	2.018 (8)	C8—H8A	0.9500
Cu2—N6^i^	2.053 (9)	C9—C10	1.371 (15)
Cu2—N5^i^	2.107 (8)	C9—H9A	0.9500
N1—C5	1.350 (12)	C10—C11	1.382 (14)
N1—C1	1.361 (13)	C10—H10A	0.9500
N2—C6	1.291 (12)	C11—H11A	0.9500
N2—C4	1.445 (12)	C12—C13	1.415 (15)
N3—C11	1.341 (13)	C12—H12A	0.9500
N3—C7	1.362 (12)	C13—C14	1.362 (15)
N4—C12	1.323 (12)	C13—H13A	0.9500
N4—C16	1.372 (13)	C14—C15	1.390 (14)
N5—C17	1.270 (12)	C14—H14A	0.9500
N5—C15	1.426 (13)	C15—C16	1.381 (13)
N5—Cu2^ii^	2.107 (8)	C16—H16A	0.9500
N6—C22	1.347 (14)	C17—C18	1.439 (15)
N6—C18	1.376 (13)	C17—H17A	0.9500
N6—Cu2^ii^	2.053 (9)	C18—C19	1.382 (14)
C1—C2	1.376 (14)	C19—C20	1.366 (16)
C1—H1A	0.9500	C19—H19A	0.9500
C2—C3	1.386 (14)	C20—C21	1.413 (15)
C2—H2A	0.9500	C20—H20A	0.9500
C3—C4	1.358 (14)	C21—C22	1.382 (16)
C3—H3A	0.9500	C21—H21A	0.9500
C4—C5	1.339 (13)	C22—H22A	0.9500
			
N4—Cu1—N3	119.2 (4)	N3—C7—C8	122.0 (10)
N4—Cu1—N2	125.5 (3)	N3—C7—C6	114.4 (9)
N3—Cu1—N2	80.4 (3)	C8—C7—C6	123.5 (10)
N4—Cu1—I1	105.3 (3)	C7—C8—C9	120.7 (11)
N3—Cu1—I1	112.1 (2)	C7—C8—H8A	119.6
N2—Cu1—I1	112.9 (2)	C9—C8—H8A	119.6
N1—Cu2—N6^i^	126.9 (3)	C10—C9—C8	118.5 (11)
N1—Cu2—N5^i^	113.9 (3)	C10—C9—H9A	120.8
N6^i^—Cu2—N5^i^	79.8 (3)	C8—C9—H9A	120.8
N1—Cu2—I2	109.9 (2)	C9—C10—C11	118.2 (11)
N6^i^—Cu2—I2	106.7 (2)	C9—C10—H10A	120.9
N5^i^—Cu2—I2	117.3 (2)	C11—C10—H10A	120.9
C5—N1—C1	118.5 (9)	N3—C11—C10	124.3 (11)
C5—N1—Cu2	121.7 (7)	N3—C11—H11A	117.8
C1—N1—Cu2	119.7 (7)	C10—C11—H11A	117.8
C6—N2—C4	122.4 (9)	N4—C12—C13	123.7 (11)
C6—N2—Cu1	111.2 (7)	N4—C12—H12A	118.2
C4—N2—Cu1	126.4 (6)	C13—C12—H12A	118.2
C11—N3—C7	116.3 (9)	C14—C13—C12	119.0 (10)
C11—N3—Cu1	131.4 (8)	C14—C13—H13A	120.5
C7—N3—Cu1	112.3 (7)	C12—C13—H13A	120.5
C12—N4—C16	116.4 (9)	C13—C14—C15	118.5 (10)
C12—N4—Cu1	122.0 (8)	C13—C14—H14A	120.8
C16—N4—Cu1	120.5 (6)	C15—C14—H14A	120.8
C17—N5—C15	121.6 (9)	C16—C15—C14	119.4 (11)
C17—N5—Cu2^ii^	111.3 (7)	C16—C15—N5	114.6 (9)
C15—N5—Cu2^ii^	126.8 (6)	C14—C15—N5	125.9 (9)
C22—N6—C18	117.7 (9)	N4—C16—C15	122.9 (9)
C22—N6—Cu2^ii^	128.6 (7)	N4—C16—H16A	118.5
C18—N6—Cu2^ii^	113.1 (7)	C15—C16—H16A	118.5
N1—C1—C2	121.3 (10)	N5—C17—C18	121.6 (10)
N1—C1—H1A	119.3	N5—C17—H17A	119.2
C2—C1—H1A	119.3	C18—C17—H17A	119.2
C1—C2—C3	118.2 (10)	N6—C18—C19	121.5 (10)
C1—C2—H2A	120.9	N6—C18—C17	113.8 (9)
C3—C2—H2A	120.9	C19—C18—C17	124.7 (10)
C4—C3—C2	119.5 (10)	C20—C19—C18	121.5 (11)
C4—C3—H3A	120.2	C20—C19—H19A	119.3
C2—C3—H3A	120.2	C18—C19—H19A	119.3
C5—C4—C3	120.5 (10)	C19—C20—C21	116.8 (11)
C5—C4—N2	124.3 (9)	C19—C20—H20A	121.6
C3—C4—N2	115.2 (9)	C21—C20—H20A	121.6
C4—C5—N1	121.8 (9)	C22—C21—C20	120.1 (11)
C4—C5—H5A	119.1	C22—C21—H21A	119.9
N1—C5—H5A	119.1	C20—C21—H21A	119.9
N2—C6—C7	121.3 (9)	N6—C22—C21	122.4 (11)
N2—C6—H6A	119.4	N6—C22—H22A	118.8
C7—C6—H6A	119.4	C21—C22—H22A	118.8

Symmetry codes: (i) −*x*+5/2, *y*+1/2, −*z*+1/2; (ii) −*x*+5/2, *y*−1/2, −*z*+1/2.

### Hydrogen-bond geometry (Å, º)

**Table:**

*D*—H···*A*	*D*—H	H···*A*	*D*···*A*	*D*—H···*A*
C22—H22*A*···I1^iii^	0.95	3.03	3.789 (12)	138
C20—H20*A*···I1^iv^	0.95	3.14	4.025 (12)	156
C17—H17*A*···I2^v^	0.95	3.16	4.011 (11)	149

Symmetry codes: (iii) *x*, *y*−1, *z*; (iv) −*x*+3, −*y*−1, −*z*+1; (v) *x*+1/2, −*y*−1/2, *z*+1/2.
